# Comparative evaluation of deep learning models for three-class frailty assessment using gait metrics

**DOI:** 10.3389/fragi.2026.1873618

**Published:** 2026-07-07

**Authors:** Charmayne Mary Lee Hughes, Yan Zhang

**Affiliations:** Age-Appropriate Human-Machine Systems, Institute of Psychology and Ergonomics, Technische Universität Berlin, Berlin, Germany

**Keywords:** ageing, deep learning, frailty, gait, IMU (inertial measurement unit), multivariate time-series classification

## Abstract

**Background:**

Frailty assessment in older adults typically relies on subjective clinical tools that are time-consuming and require trained personnel, limiting their use in routine or large-scale screening. Wearable sensor-based gait analysis offers a promising objective alternative; however, comprehensive evaluations of deep learning models for multi-class frailty classification using structured gait metrics remain limited.

**Methods:**

This study evaluates multiple deep learning architectures for three-class frailty classification using structured gait features derived from a single wearable inertial measurement unit (IMU). Four architectures (Transformer, ShapeFormer, InceptionTime, and LSTM-CNN) were evaluated using the publicly available GSTRIDE dataset, comprising 163 older adults (non-frail: *n* = 65, pre-frail: *n* = 77, frail: *n* = 21). Eleven clinically interpretable stride-level gait variables, extracted from a single IMU, were used as model input. A 10-fold participant-level cross-validation scheme, matching the default configuration of the original implementation (cv_seed = 1), was applied to ensure robust performance estimation and prevent data leakage.

**Results:**

The four architectures yielded comparable mean accuracy under a unified protocol (ShapeFormer 73.1% ± 10.6%, Transformer 72.6% ± 6.0%, InceptionTime 70.5% ± 9.5%, LSTM-CNN 65.9% ± 9.8%). A Friedman omnibus test on the per-fold scores did not reject the null of equal performance (borderline on accuracy and macro-F1 (*p* = 0.050 and 0.056 respectively), with no significant difference for macro-AUC [*p* = 0.840]), indicating that the apparent ranking is within sampling variability. Performance was higher for non-frail and pre-frail individuals, while classification of frail individuals remained challenging across all models.

**Conclusion:**

Multi-class frailty classification using structured gait metrics from a single wearable sensor is feasible, though performance varies across models and frailty classes. Model stability and sensitivity to specific classes represent important trade-offs.

**Significance:**

These findings support the development of scalable, objective approaches to frailty assessment and underscore the importance of selecting models based on clinical priorities.

## 1 Introduction

The global population is aging at an unprecedented rate. The number of individuals aged 65 years and older is projected to increase substantially in the coming decades (Jakovljevic et al., 2021), placing growing demands on healthcare systems due to rising prevalence of chronic disease, functional decline, and geriatric syndromes. Among these, frailty has emerged as a critical public health concern because of its strong association with adverse outcomes and its potential reversibility when identified early (Clegg et al., 2013).

Frailty is a multidimensional syndrome characterized by reduced physiological reserve and increased vulnerability to stressors (Fried et al., 2001), affecting approximately 13%–23.2% of community-dwelling older adults (Cai et al., 2025). It is associated with increased risk of falls, hospitalization, disability, and mortality (Dent et al., 2019; 2024). Importantly, frailty exists on a continuum, with the intermediate pre-frail state representing a critical window for intervention. Identifying pre-frailty at an early stage enables targeted strategies, including exercise and nutritional interventions that may delay or reverse progression to frailty (Dent et al., 2019). However, effective early identification depends on reliable and scalable methods for assessing frailty in both clinical and real-world settings.

Current clinical assessments, such as the Fried Frailty Phenotype (Fried et al., 2001) and the Clinical Frailty Scale (Rockwood et al., 2005), are widely used but have important limitations. The Fried Frailty Phenotype requires multiple physical measurements and trained personnel, making it relatively time-consuming and less feasible for routine or large-scale screening, while the Clinical Frailty Scale relies on clinical judgement and may be subject to inter-rater variability. In addition, frailty is typically assessed intermittently in clinical settings, which may reduce sensitivity to gradual functional decline over time (Dent et al., 2019). These limitations highlight the need for objective, scalable approaches that can support continuous or repeated assessment of frailty.

Gait has long been recognized as a sensitive marker of health and functional status in older adults (Studenski et al., 2011; De la Cámara et al., 2020). Alterations in gait reflect impairments in neuromuscular control, balance, and energy regulation, all of which are central components of the frailty phenotype. Parameters such as gait speed, variability, and cadence have been consistently associated with frailty and risk of adverse outcomes (Studenski et al., 2011; Verghese and Xue, 2011). Wearable sensing technologies, particularly inertial measurement units (IMUs), enable objective quantification of these gait characteristics in real-world settings and have emerged as a promising tool for mobility assessment (Muro-De-La-Herran et al., 2014; Schwenk et al., 2015).

While prior work has applied machine learning to IMU-based frailty detection, several limitations remain. Many studies focus on binary classification (Lien et al., 2025), overlooking the clinically important distinction between non-frail, pre-frail, and frail individuals. Multi-class classification is more clinically meaningful but substantially more challenging (Hughes and Zhang, 2026; Griškevičius, et al., 2025). However, even in multi-class settings, performance is often reported only in aggregate terms, with limited or no class-specific evaluation, making it difficult to assess performance on clinically critical minority classes such as frail individuals. In addition, data leakage (i.e., where data from the same participant appear in both training and test sets) remains a methodological concern in IMU-based gait studies, particularly in settings where multiple correlated stride-level samples are derived from individual participants (Amjad et al., 2025). Finally, many approaches rely on raw or minimally processed sensor signals, which may limit clinical interpretability, reducing their transparency to clinicians and potentially limiting adoption in clinical practice (Topol, 2019).

Structured gait metrics provide an alternative representation that is both clinically interpretable and readily derived from wearable sensors (Muro-De-La-Herran et al., 2014). At the same time, advances in deep learning offer the ability to model complex temporal relationships within such data (Zhang et al., 2022). However, the relative performance of modern deep learning architectures for multi-class frailty classification using structured gait metrics remains unclear, particularly under rigorous evaluation conditions. Therefore, this study presents a systematic comparison of multiple deep learning architectures for three-class frailty classification using gait metrics derived from a single wearable IMU during walking. The analysis is conducted using the publicly available GSTRIDE dataset (García-de-Villa et al., 2023), and participant-level cross-validation is applied to ensure robust and unbiased performance estimation.

The contributions of this study are threefold. First, it provides a systematic comparison of several deep learning architectures, including Transformer, ShapeFormer, InceptionTime, and LSTM-CNN, for multi-class frailty classification. Second, it addresses a key methodological limitation in prior IMU-based gait studies by enforcing strict participant-level data separation to mitigate data leakage. Third, it demonstrates the feasibility of using clinically interpretable gait metrics, rather than raw sensor signals, to support scalable and objective frailty assessment.

## 2 Methods

### 2.1 Dataset

The GSTRIDE database was used, a publicly available dataset containing gait measurements from community-dwelling older adults residing in Spain (García-de-Villa et al., 2023). The dataset comprises 163 participants (45 men, 118 women) aged 70–98 years (mean = 82.6, SD = 6.2). Participants were classified according to the Fried Frailty Phenotype (Fried et al., 2001), which assesses five criteria: unintentional weight loss, self-reported exhaustion, low physical activity, slow walking speed, and weak grip strength. Based on these criteria, participants were categorized as non-frail (0 criteria; *n* = 65, 39.9%), pre-frail (1-2 criteria; *n* = 77, 47.2%), or frail (≥3 criteria; *n* = 21, 12.9%). All participants provided written informed consent, and the study was approved by the relevant ethics committee (Ethics approval: PI 4486).

Gait measurements were recorded using a 9-DoF IMU comprising tri-axial accelerometers, gyroscopes, and magnetometers, implemented either using a Physiolog 6 S system or a custom-built platform based on an LSM6DSRX IMU. Each IMU was affixed to the dorsum of the participant’s preferred foot using elastic straps. Participants walked at a self-selected comfortable pace in a controlled indoor environment for approximately 30 min. Recording durations varied across participants (5 - >30 min), reflecting natural variability in trial completion times.

### 2.2 Feature extraction and data representation

Each participant’s gait recording was processed into a sequence of stride-level feature vectors. For every detected stride, 11 clinically interpretable variables were extracted (Table 1): six temporal parameters (stride time, swing, toe-off, heel-strike and foot-flat percentages of the gait cycle, cadence), three spatial parameters (speed, stride length, clearance), and two kinematic parameters (toe-off angle, heel-strike angle). All variables are taken directly from the stride-level metric files distributed with GSTRIDE (García-de-Villa et al., 2023). No aggregation across strides was performed prior to model input; per-stride values were preserved as a sequence to allow temporal models to exploit within-recording dynamics. The resulting input for each participant *i* is a two-dimensional array of shape (*T*_*i*_, 11), where *T*_*i*_ is the number of detected strides. Across the 163 participants, *T*_*i*_ ranged from 213 to 2,185 strides (median 1,064; 25th–75th percentile 793–1,399).

**TABLE 1:** Stride-level variables extracted from the GSTRIDE dataset and used as model inputs. Variables comprise six temporal gait parameters, three spatial gait parameters, and two kinematic gait parameters. Values were retained at the individual stride level and provided to the models as sequential observations.

#	Variable	Unit	Category	Definition
1	Stride time	s	Temporal	Time between two successive heel strikes of the same foot
2	Swing	% Gait cycle	Temporal	Proportion of the gait cycle spent in swing phase
3	Toe off	% Gait cycle	Temporal	Gait-cycle percentage at which toe-off occurs
4	Heel strike	% Gait cycle	Temporal	Gait-cycle percentage at which heel strike occurs
5	Foot flat	% Gait cycle	Temporal	Proportion of gait cycle with the foot flat on the ground
6	Toe-off angle	°	Kinematic	Foot pitch angle at toe-off
7	Heel-strike angle	°	Kinematic	Foot pitch angle at heel strike
8	Cadence	strides·min^-1^	Temporal	Instantaneous stride rate
9	Speed	m·s^-1^	Spatial	Instantaneous walking speed
10	Stride length	m	Spatial	Distance between two successive same-foot heel strikes
11	Clearance	m	Spatial	Maximum vertical foot clearance during swing

Within each mini-batch, sequences were zero-padded to the length of the longest sequence in the batch. No truncation, windowing, or fixed-window subsampling was performed for the Transformer, InceptionTime, and LSTM-CNN models. Because ShapeFormer relies on absolute positional indexing, input sequences were linearly resampled to a fixed length of 512 strides per participant before model input.

### 2.3 Preprocessing

The distributed GSTRIDE metric files were inspected and found to contain no missing values; consequently, no imputation was required. For each cross-validation fold, a RobustScaler was fitted exclusively on the training data and subsequently applied to the corresponding validation sequences without re-fitting. Thus, all preprocessing parameters were derived solely from the training subset, preventing information leakage from the validation data.

### 2.4 Deep learning architectures

Four representative deep learning models spanning different paradigms in time-series classification were evaluated: an attention-based Transformer, a shapelet-based ShapeFormer, a multi-scale convolutional InceptionTime model, and a hybrid LSTM-CNN architecture.

The Transformer (Vaswani et al., 2017) served as the baseline model, representing the current state of the art in sequence modeling. The implementation followed a standard encoder-only architecture with the following specifications: (1) input embedding via linear projection of 11-dimensional gait features into a 128-dimensional latent space (2) sinusoidal positional encoding to preserve temporal order; (3) four stacked encoder blocks, each comprising multi-head self-attention (8 heads) and position-wise feedforward networks (hidden dimension 512); and (4) a classification head consisting of global average pooling followed by a linear projection to three output classes. The self-attention mechanism computes attention weights as:

$$Attention\left(Q,K,V\right)=softmax\left(\frac{{QK}^{T}}{\sqrt{d_{k}}}\right)\;V$$

where *Q*, *K*, and *V* denote the query, key, and value matrices, respectively, and *dk* is the key dimensionality.

*ShapeFormer* (Le et al., 2024) integrates shapelet discovery with transformer-based attention mechanisms. The architecture consists of two parallel processing streams. The class-specific shapelet stream identifies discriminative subsequences (shapelets) that characterize each frailty class. For each class c, the shapelet discovery process identifies subsequences *S*_*c*_ = {*s*_*1*_ *, s*_*2*_, … , *s*_*k*_} that maximize information gain for distinguishing class c from the remaining classes. The resulting shapelet transform computes distances between input sequences and the discovered shapelets, yielding a feature representation that captures class-discriminative temporal patterns. In parallel, the generic feature stream employs a one-dimensional convolutional network with three convolutional layers (kernel sizes 3, 5, and 7) to extract class-agnostic temporal features. The outputs of both streams are processed by separate transformer encoders and subsequently fused via concatenation, followed by a classification layer. This dual-stream design enables the model to jointly leverage class-specific discriminative patterns and general temporal representations.

*InceptionTime* (Fawaz et al., 2020) adapts the Inception architecture from computer vision to time-series classification. Its core design employs parallel convolutional filters with multiple kernel sizes within each Inception module. Specifically, each module applies convolutions with kernel sizes of 10, 20, and 40 in parallel, along with a max-pooling branch, enabling the model to capture temporal patterns at multiple scales simultaneously. Residual connections between modules facilitate gradient propagation and enable the training of deeper architectures. The final model is constructed as an ensemble of five independently trained networks, with predictions averaged to reduce variance and improve robustness.

The *LSTM–CNN hybrid architecture* combines the sequential modeling capabilities of recurrent networks with the local feature extraction of convolutional layers. The model consists of a 128-unit bidirectional LSTM layer that captures both forward and backward temporal dependencies, followed by two one-dimensional convolutional layers (64 and 32 filters, kernel size 3) that extract local patterns from the LSTM outputs. Dropout (rate = 0.3) and batch normalization are applied for regularization. The classification head comprises global average pooling followed by a dense layer with softmax activation.

### 2.5 Experimental setup

#### 2.5.1 Cross-validation strategy

A 10-fold stratified cross-validation strategy was employed at the participant level (Kohavi, 1995), matching the default configuration of the original Hughes and Zhang (2026) implementation (cv_seed = 1; 80/20 stratified holdout with 33 participants reserved for future external validation, followed by stratified K-fold cross-validation with *K* = 10 on the 130-participant development set). This design ensures that all measurements from a given participant appear exclusively in either the training or validation set, never both, preventing data leakage whereby a model could learn participant-specific patterns rather than generalizable frailty indicators. Each outer fold provided approximately 117 participants for training and 13 for validation, with stratification ensuring proportional representation of all three frailty classes in each split. Across the ten folds the full development cohort appeared in validation exactly once.

#### 2.5.2 Training configuration

All models were implemented in PyTorch. The Transformer, InceptionTime and LSTM-CNN baselines were trained using AdamW (Loshchilov and Hutter, 2019) with a learning rate of 1 × 10^−3^, weight decay 1 × 10^−4^, a CosineAnnealingLR schedule (*T*_max_ = 50, *η*_min_ = 1 × 10^−6^), global *ℓ*_*2*_ gradient clipping at 1.0, and a mini-batch size of 32. ShapeFormer was trained using the upstream defaults of Le et al. (2024) (i.e., RAdam, mini-batch = 8, dropout = 0.4) while retaining the same cross-validation and class-weighting procedures. All models were trained for a maximum of 200 epochs with early stopping (patience = 20) based on validation accuracy. Within each outer fold, the held-out validation partition was used both for early stopping and for final performance estimation (e.g., Fawaz et al., 2020; Wang et al., 2017). The implications of this design are discussed in the Limitations section. Model evaluation followed a unified protocol comprised ten stratified outer cross-validation folds and three random seeds (123, 456, 789), resulting in 30 runs per architecture. The outer cross-validation partition was fixed (cv_seed = 1, matching the default specified in the original implementation), such that variation across runs reflected only differences in model initialization and training stochasticity. InceptionTime was implemented as the canonical five-network ensemble described by Fawaz et al. (2020), with predictions averaged across ensemble members at inference time.

#### 2.5.3 Hyperparameter selection

Architecture hyperparameters were adopted directly from the original publications of the respective architectures (Vaswani et al., 2017; Le et al., 2024; Fawaz et al., 2020; and the LSTM-CNN specification in the manuscript text) without further tuning, to avoid biased model selection on a small sample. Training hyperparameters were fixed from initial debugging on the training partitions only and were not optimized on the validation folds; the complete configuration is reported in Supplementary Table S1. Because no formal hyperparameter search was conducted, the reported per-model results should be interpreted as the performance of a single reasonable configuration rather than a tuned upper bound.

#### 2.5.4 Class imbalance handling

To address the inherent class imbalance in the dataset, class-weighted cross-entropy loss was employed. The weight for each class was computed as inversely proportional to its frequency:

$$\omega_{c}=\frac{N}{C\;x\;n_{c}}$$

where *N* is the total number of participants in the training fold, *C* is the number of classes (3), and *n*_*c*_ is the number of training-fold participants belonging to class *c*. Because each participant contributes exactly one gait sequence to the model, participants—not individual strides—constitute the training samples used in the weighting calculation. This weighting scheme ensures that minority classes, particularly the frail category, receive proportionally greater importance during training.

### 2.6 Evaluation metrics

#### 2.6.1 Model performance

Model performance was evaluated using multiple complementary metrics to provide a comprehensive assessment of classification accuracy across frailty categories. Overall accuracy was reported as the proportion of correctly classified instances. To account for class imbalance, macro-averaged precision was computed as the unweighted mean of precision across all classes. The weighted F1-score was used to balance precision and recall, with scores weighted by class support. Macro-averaged area under the receiver operating characteristic curve (ROC AUC) was calculated using a one-vs-rest approach for each class. Per-class precision, recall, and F1-score were also reported for each frailty category to enable detailed interpretation of model performance. All metrics were averaged across ten cross-validation folds × three random seeds (30 paired observations per model) and presented as mean ± standard deviation.

#### 2.6.2 Statistical analysis

To assess whether the observed differences in mean performance among the four architectures exceeded what could be expected from sampling variability, a Friedman omnibus test was performed on the ten participant-level cross-validation folds, with results averaged across random seeds within each fold and the fold treated as the unit of statistical comparison (Demšar, 2006). Post-hoc comparisons were conducted using the Nemenyi test (Demšar, 2006) and paired t-tests with Holm–Bonferroni correction (Holm, 1979). Because ten paired observations were available per architecture, statistical power was limited. Consequently, a failure to reject the null hypothesis should not be interpreted as evidence of equivalence between architectures. Rather, the results should be interpreted as reflecting the strength of evidence available given the size and structure of the present dataset.

## 3 Results

### 3.1 Overall and multi-metric performance

Table 2 summarises the aggregate performance of all four architectures in three-class frailty classification under the unified evaluation protocol described in Methods. Overall accuracy ranged from 65.9% (LSTM-CNN) to 73.1% (ShapeFormer), with Transformer at 72.6% and InceptionTime at 70.5%. Transformer exhibited the lowest fold-to-fold variability in accuracy (±6.0%), while the other three architectures showed wider spread (±9.5% to ±10.6%). Differences in mean accuracy were modest and, as detailed below, were at the borderline of significance under a Friedman omnibus test followed by Holm-Bonferroni correction.

**TABLE 2:** Classification performance for multi-class frailty prediction (non-frail, pre-frail, frail) using structured gait metrics derived from a single IMU.

Model	Accuracy	Weighted F1-Score	Macro F1-Score	Macro-averaged ROC AUC
Transformer	72.6 ± 6.0	72.2 ± 6.0	68.4 ± 12.6	76.3 ± 8.9
InceptionTime	70.5 ± 9.5	69.8 ± 10.0	70.3 ± 12.8	77.7 ± 11.5
LSTM-CNN	65.9 ± 9.8	64.7 ± 12.2	64.5 ± 11.6	76.1 ± 7.5
ShapeFormer	73.1 ± 10.6	72.0 ± 11.8	71.5 ± 12.2	79.0 ± 9.6

Differences in discriminative ranking ability were relatively modest across models. ShapeFormer achieved the highest macro-averaged ROC AUC (79.0% ± 9.6%), followed by InceptionTime (77.7% ± 11.5%), Transformer (76.3% ± 8.9%), and LSTM-CNN (76.1% ± 7.5%). The 2.9 percentage-point range of macro-AUC values, smaller than the per-architecture standard deviations (±7.5% to ±11.5%), indicates that the ranking ability of the four architectures is statistically indistinguishable under this protocol, consistent with the omnibus Friedman test result (*p* = 0.840 for macro-AUC) reported below.

The Friedman omnibus test was performed on per-fold scores after collapsing across the three model seeds, yielding *n* = 10 paired observations per architecture (Supplementary Table S2). The test statistics were *χ* ^*2*^ (3) = 7.80, *p* = 0.050 for accuracy; *χ* ^*2*^ (3) = 7.56, *p* = 0.056 for macro F1-score; *χ* ^*2*^ (3) = 0.84, *p* = 0.840 for macro-AUC; *χ* ^*2*^ (3) = 3.78, *p* = 0.286 for frail-class recall; and *χ* ^*2*^ (3) = 3.95, *p* = 0.267 for frail-class F1-score. The accuracy and macro F1-score statistics were at, or only marginally above, the conventional *α* = 0.05 threshold, providing at most weak evidence that the four architectures differ in overall classification performance. Consistent with this result, Holm-corrected pairwise comparisons did not identify any statistically significant differences between individual architecture pairs. Given the limited number of paired observations (ten per architecture), the omnibus test was sensitive only to relatively large effects. Consequently, the architectural comparison is framed primarily in terms of class-specific trade-offs and fold-to-fold stability rather than overall ranking.

Taken together, the four architectures span a 7.2 percentage-point range in mean accuracy (65.9%–73.1%), and this range is comparable in magnitude to the per-fold standard deviations of each architecture (±6.0% to ±10.6%). Differences in overall ranking are therefore consistent with sampling variability, and the architectural comparison is positioned as one of per-class trade-offs (see Per-Class Performance below).

### 3.2 Per-class performance

Table 3 presents per-class precision, recall, and F1-scores across all models. Per-fold strip and box plots that expose the within-architecture fold-to-fold spread for the frail class are provided in Supplementary Figure S1 (structured input) and Supplementary Figure S2 (raw IMU input). A consistent pattern is observed for the frail class, which shows the lowest performance and highest variability across models. However, the relative performance between the non-frail and pre-frail classes varies by model, indicating model-dependent differences in class-level performance.

**TABLE 3:** Per-class performance across models. Precision, recall, and F1-score are shown for each frailty class and model.

Model	Class	Precision	Recall	F1-score
Transformer	Non-frail	81.8 ± 13.7	70.8 ± 13.1	74.7 ± 9.0
	Pre-frail	74.4 ± 11.0	76.0 ± 13.5	73.8 ± 6.5
	Frail	55.2 ± 38.6	63.3 ± 41.4	56.8 ± 37.2
InceptionTime	Non-frail	74.3 ± 17.0	65.0 ± 17.1	67.8 ± 13.1
	Pre-frail	72.3 ± 12.7	71.6 ± 17.9	70.1 ± 11.4
	Frail	71.7 ± 33.1	81.7 ± 33.4	73.0 ± 29.5
LSTM-CNN	Non-frail	76.7 ± 19.2	60.0 ± 20.6	63.8 ± 16.7
	Pre-frail	69.3 ± 18.0	66.8 ± 24.5	66.3 ± 19.1
	Frail	55.3 ± 28.4	85.0 ± 26.7	63.3 ± 24.1
ShapeFormer	Non-frail	79.1 ± 17.9	63.1 ± 17.9	68.1 ± 13.3
	Pre-frail	76.6 ± 15.1	80.2 ± 21.7	76.2 ± 15.9
	Frail	67.8 ± 30.9	81.7 ± 30.7	70.2 ± 26.3

The frail class, which is of particular clinical relevance for early intervention, exhibited the greatest fold-to-fold variability across all architectures. Mean frail-class F1-scores were highest for InceptionTime (73.0% ± 29.5%), followed by ShapeFormer (70.2% ± 26.3%) and LSTM-CNN (63.3% ± 24.1%), whereas the Transformer achieved the lowest mean frail-class F1-score (56.8% ± 37.2%). LSTM-CNN additionally demonstrated the highest frail-class recall (85.0% ± 26.7%), suggesting a more recall-oriented position on the precision–recall trade-off. The large standard deviations are a direct consequence of the limited number of frail participants available for evaluation. With only 21 frail participants distributed across ten stratified validation folds, each fold contains approximately one to two frail individuals. Under these conditions, a single misclassification can substantially affect fold-level performance estimates, resulting in high variability across folds. Accordingly, pairwise differences in frail-class F1-scores did not remain significant after Holm correction and should be interpreted cautiously given the present sample size.

Across all models, performance in the pre-frail class is generally higher than in the frail class, although its relative ordering with respect to the non-frail class varies across models. Differences between models are generally less pronounced for the pre-frail class than for the frail class, although variability remains evident in some cases.

### 3.3 Ablation studies

To better characterize which design choices materially affect the reported performance, we conducted two controlled ablations under the unified evaluation protocol. The first compares the primary class-weighted cross-entropy criterion against focal loss; the second compares the structured stride-level representation against raw IMU input. In both ablations the CV partition, hyperparameters, model seeds and class-weighting scheme are held fixed, isolating the effect of the manipulated component.

#### 3.3.1 Loss function and threshold sensitivity

The primary class-weighted cross-entropy objective was compared with focal loss (*γ* = 2.0; *α* equal to the participant-level inverse-frequency class weights) under the same cross-validation protocol as the main experiments (Supplementary Table S3; 120 additional runs). The direction and magnitude of the effect depended on the architecture. Overall accuracy under focal loss decreased by 1.0–2.3 percentage points for the Transformer (72.6% vs. 70.8%), InceptionTime (70.5% vs. 69.5%), and LSTM-CNN (65.9% vs. 63.6%), while increasing by 0.7 percentage points for ShapeFormer (73.1% vs. 73.8%). Changes in frail-class F1-score were mixed: focal loss reduced performance by 4.1 percentage points for the Transformer (56.8% vs. 52.7%) and by 6.5 percentage points for ShapeFormer (70.2% vs. 63.7%), but increased performance by 2.2 percentage points for LSTM-CNN (63.3% vs. 65.5%) and by 0.3 percentage points for InceptionTime (73.0% vs. 73.3%). Because no consistent advantage emerged and the two highest-accuracy architectures lost frail-class performance under focal loss, class-weighted cross-entropy was retained as the primary objective; focal-loss results are reported to document the sensitivity of the findings to this design choice.

Frail-class precision–recall curves were additionally examined (Supplementary Figure S3), together with clinically motivated operating points (Supplementary Table S4). Average precision for the frail class ranged from 0.47 (Transformer) to 0.74 (InceptionTime), substantially exceeding the random baseline of 0.13. At an operating point selected to achieve a precision of at least 0.50, InceptionTime achieved a frail-class precision of 55% ± 15% with a recall of 85% ± 33%, compared with 72% ± 33% precision and 82% ± 33% recall under the default argmax decision rule, indicating that the default operating point already trades aggressively for recall.

#### 3.3.2 Raw IMU versus structured features

To isolate the contribution of structured features, we repeated the analysis with raw IMU inputs (linearly resampled to 4,096 samples per participant) under the identical CV partition, identical hyperparameters, identical class-weighting scheme and identical model architectures (Table 4). The raw IMU comparison uses *n* = 158 participants, as raw IMU recordings were unavailable for five of the 163 participants in the original dataset (García-de-Villa et al., 2023).

**TABLE 4:** Controlled ablation comparing raw IMU input against the structured stride-level representation. The same CV partition, hyperparameters, class-weighting scheme and model architectures are used for both input regimes.

Model	Input	Accuracy	Macro F1-score	Frail-class F1-Score
Transformer	Raw IMU (n = 158)	63.3 ± 8.6	56.8 ± 13.1	44.4 ± 36.7
	Structured (n = 163)	72.6 ± 6.0	68.4 ± 12.6	56.8 ± 37.2
InceptionTime	Raw IMU (n = 158)	68.5 ± 9.9	64.1 ± 16.6	59.6 ± 38.8
	Structured (n = 163)	70.5 ± 9.5	70.3 ± 12.8	73.0 ± 29.5
LSTM-CNN	Raw IMU (n = 158)	62.4 ± 11.6	57.1 ± 19.9	51.9 ± 31.7
	Structured (n = 163)	65.9 ± 9.8	64.5 ± 11.6	63.3 ± 24.1
ShapeFormer	Raw IMU (n = 158)	63.0 ± 10.0	56.5 ± 17.7	46.3 ± 41.1
	Structured (n = 163)	73.1 ± 10.6	71.5 ± 12.2	70.2 ± 26.3

The structured representation improved classification accuracy across all four architectures: the Transformer (63.3% vs. 72.6%, +9.3 percentage points), ShapeFormer (63.0% vs. 73.1%, +10.1 percentage points), LSTM-CNN (62.4% vs. 65.9%, +3.5 percentage points) and InceptionTime (68.5% vs. 70.5%, +2.0 percentage points). Macro F1-scores improved correspondingly, with the largest gains observed for Transformer (+11.6 percentage points) and ShapeFormer (+15.0 percentage points). Frail-class F1-scores showed consistent improvement under structured input for all four models (Transformer +12.4 percentage points, InceptionTime +13.4 percentage points, LSTM-CNN +11.4 percentage points, ShapeFormer +23.9 percentage points), albeit with wide fold-to-fold variability driven by the small frail class support. Overall, these findings indicate that the structured stride-level representation confers a meaningful and consistent performance advantage relative to raw IMU input across all four architectures.

## 4 Discussion

This study presents a systematic comparison of deep learning models for three-class frailty assessment using structured gait metrics derived from a single IMU. The overall performance across models ranged from 65.9% to 73.1% accuracy, reflecting the inherent difficulty of distinguishing between non-frail, pre-frail, and frail states using gait features alone. This moderate performance is particularly notable given the high degree of overlap in gait patterns between adjacent frailty categories, especially between pre-frail and frail individuals, who often exhibit subtle but clinically meaningful changes in movement dynamics. The small sample size of the frail class (12.9% of the cohort) further complicates classification, as models may struggle to learn robust representations from limited training data. Despite these results, the observed performance should be interpreted cautiously, particularly given the moderate sensitivity for the frail class, which represents the most clinically critical group. Misclassification in this group has greater clinical implications, as frail individuals are at the highest risk of adverse outcomes and may benefit most from early intervention.

Given the clinical importance of detecting frailty early, and the challenges posed by subtle gait changes and class imbalance, the observed performance improvement suggests that structured feature engineering enhances model reliability. The current approach, which uses structured gait metrics, achieves an overall accuracy of up to 73.1% (ShapeFormer) and a best frail-class F1-score of 73.0% (InceptionTime). A controlled within-study ablation (Table 4) shows that structured features confer a consistent +2.0 to +10.1 percentage-point accuracy improvement across all four architectures over raw IMU input under an otherwise identical protocol (Transformer +9.3, ShapeFormer +10.1, LSTM-CNN +3.5, InceptionTime +2.0). The contrast with our prior work (Hughes and Zhang, 2026) (raw-IMU InceptionTime frail-class F1-score = 24.9%) was confounded by changes in the cross-validation scheme, hyperparameters and class-weighting; the present ablation isolates the input-only effect. This improvement demonstrates that transforming raw sensor data into clinically meaningful gait parameters enhances model performance and supports more reliable frailty assessment. The use of structured gait metrics offers several advantages over raw sensor signals. First, these features are directly interpretable and clinically meaningful, aligning with established gait parameters used in physical assessments. This enables clinicians to understand and trust model outputs, bridging the gap between AI and clinical practice. Second, structured features reduce computational complexity and enable deployment in low-latency, resource-constrained environments such as primary care clinics and home monitoring systems.

Although overall accuracy is comparable across the four architectures, per-class profiles diverge in clinically meaningful ways. ShapeFormer combines the highest overall accuracy (73.1%) and macro-AUC (79.0%) with competitive frail class performance (frail-class F1-score = 70.2%), positioning it as a balanced default when no class is prioritised. InceptionTime delivers the highest frail-class F1-score (73.0%, frail-class recall 81.7%) and is therefore preferable when sensitivity to the frail class is the clinical priority. The Transformer exhibits the lowest accuracy variance (±6.0%) - roughly half that of the other architectures - indicating more stable behaviour across folds and a possibly tighter risk envelope at deployment. LSTM-CNN attains the highest frail recall (85.0%) but with the lowest overall accuracy (65.9%), suggesting that its decision boundary trades aggressively for sensitivity. Because no pairwise difference reaches Holm-corrected statistical significance, the choice among architectures should be guided by which class-level operating characteristic best matches the clinical priority rather than by a single aggregate accuracy figure.

Methodologically, the implementation of participant-level cross-validation is a key strength of this study. It prevents data leakage by ensuring that no data from a given individual appears in both training and testing sets, thereby providing a more realistic estimate of model performance. This approach addresses a common limitation in prior studies, where improper splitting of time-series data led to overly optimistic performance metrics (Amjad et al., 2025). By using structured features derived from a single IMU, this work also advances the feasibility of scalable, low-cost frailty screening, which is critical for population-level health monitoring.

The clinical implications of this work are significant. Reliable three-class classification from a single wearable sensor enables early detection of pre-frail individuals before irreversible functional decline occurs. This window of opportunity supports timely interventions such as strength training, balance programs, or nutritional support, which have been shown to delay or even reverse frailty progression. The ability to perform such assessments in non-clinical settings, such as homes or community centers, could transform frailty screening from a reactive to a proactive public health strategy.

Several limitations should be considered when interpreting the present findings. First, within each cross-validation fold the held-out validation partition was used both for early stopping and for final performance estimation. While common in small-sample deep learning (Vabalas et al., 2019), this design may introduce a degree of optimism bias relative to nested cross-validation, in which an inner split is reserved for early stopping (Bates et al., 2024); we did not adopt a nested scheme here because the resulting inner-fold size (≈12 participants) would have provided unreliable early-stopping signals. Second, the frail subgroup is small (*n* = 21), and under 10-fold stratified cross-validation each validation partition contains only one to two frail participants. This support dominates the standard deviations reported for frail-class F1-score (Table 3) and is the principal reason why pairwise comparisons fail to reach Holm-corrected significance, rather than a true absence of architectural differences. Third, the analysis is based on a single European cohort and a single walking task (García-de-Villa et al., 2023); external validation on geographically diverse cohorts and varied gait conditions is required before conclusions regarding clinical generalizability can be drawn.

Additional methodological considerations should also be noted. The raw-IMU comparison was restricted to 158 participants because raw IMU recordings were unavailable for five participants in the original dataset, whereas the structured-feature analysis used the full cohort. Finally, no formal hyperparameter optimization was performed. Architecture and training configurations were adopted from the original publications and reference implementations in order to reduce the risk of overfitting model selection to a small dataset. Accordingly, the reported results should be interpreted as the performance of representative configurations rather than as optimized upper-bound estimates.

Additionally, the current analysis is based on a single structured walking task, which may not capture the full variability of gait under different conditions, such as walking at different speeds, over longer distances, or in complex environments. Future work should incorporate larger, more diverse cohorts, longitudinal data to assess model stability over time, and multimodal sensor inputs that capture information from different biological systems and at multiple physiological levels. This includes combining gait metrics with signals such as heart rate variability, respiratory measures, and neuromuscular activity, reflecting the multisystem physiological basis of frailty described in Taylor et al. (2023). Integrating data across these domains (i.e., spanning biomechanical, cardiovascular, and neuromuscular systems) may provide a more comprehensive and physiologically grounded representation of frailty, enhancing model robustness, interpretability, and clinical relevance.

Class imbalance remains the dominant constraint on frail-class performance. The present study evaluated focal loss as an alternative to class-weighted cross-entropy. While focal loss improved overall accuracy for several architectures, its effects on frail class performance were inconsistent. Frail-class F1-score decreased for the Transformer and ShapeFormer but increased modestly for InceptionTime and LSTM-CNN. Sequence-level minority oversampling (e.g., time-series-adapted SMOTE variants, or diffusion-based augmentation) and threshold tuning beyond the operating points reported here are therefore the more promising avenues for further improving frail-class sensitivity without requiring additional labelled data. Future work should also investigate frailty-specific model extensions, such as self-supervised pretraining, hybrid CNN-Transformer models, temporal convolutional networks, and attention- or physiology-guided architectures that incorporate clinically meaningful gait phases or frailty-related feature groups. Finally, domain adaptation methods such as Domain-Adversarial Neural Network (DANN) (Ganin et al., 2016) or CORrelation ALignment (CORAL) (Sun and Saenko, 2016) could support generalization across cohorts with different demographic or sensor characteristics, enabling smaller domain-specific datasets to be combined without compromising participant privacy. Together, these directions could support the development of more robust and clinically deployable frailty screening tools.

In conclusion, this study presents a systematic comparison of four deep learning architectures for three-class frailty classification using structured gait metrics from a single wearable IMU. Under a unified evaluation protocol (10-fold stratified cross-validation, three model seeds, *n* = 30 runs per architecture), Friedman omnibus tests indicated weak evidence for differences in accuracy (*p* = 0.050) and macro F1-score (*p* = 0.056), and no evidence for differences in macro-AUC (*p* = 0.840); no pairwise comparison survived Holm correction. The four architectures therefore differ predominantly in their per-class operating characteristics rather than in overall ranking: ShapeFormer attained the highest overall accuracy (73.1%) and macro-AUC (79.0%); InceptionTime attained the highest frail-class F1-score (73.0%); the Transformer attained the lowest fold-to-fold accuracy variance (±6.0%); and LSTM-CNN attained the highest frail recall (85.0%) at the cost of lower overall accuracy. These results support the use of structured gait metrics for objective frailty assessment and indicate that the choice of architecture should be guided by the per-class operating characteristic best matched to the clinical use case.

## Data Availability

Publicly available datasets were analyzed in this study. This data can be found here: https://zenodo.org/records/6883292.
