# Supplementary material for: Comparative evaluation of deep learning models for three-class frailty assessment using gait metrics
Source: Front Aging. 2026 Jul 7;7:1873618. doi: 10.3389/fragi.2026.1873618 (PMC13385184; doi:10.3389/fragi.2026.1873618)
Supplement: Supplementary file 1 [file Table1.docx]

# **Supplementary Material**

**Supplementary Table S1**. Complete hyperparameter specification used for all four architectures across every reported run.

**Supplementary Table S2**. Friedman omnibus and Holm-corrected paired t-tests across the ten participant-level folds (seeds collapsed to per-fold averages). *n* = 10 paired observations per pairwise comparison.

**Supplementary Table S3**. Focal loss vs class-weighted cross-entropy, 30 runs per (model × criterion) under the identical CV protocol.

**Supplementary Table S4**. Frail-class precision-recall summary and threshold-tuned operating points per architecture (mean ± SD across the 30 outer-fold operating points).

**Supplementary Figure S1**. Per-fold frail-class precision / recall / F1 across the 30 (3 seeds × 10 folds) runs per architecture, structured features.

**Supplementary Figure S2**. Same as Supplementary Figure S1 but for raw IMU input (*n* = 158 participants). ShapeFormer was retrained on raw IMU using its internal resampling pipeline, allowing direct comparison with the other architectures.

**Supplementary Figure S3**. Frail-class precision-recall curves under focal loss, pooled across the 30 (seed × fold) runs per model. Average precision (AP) is shown in the legend; random baseline = 0.13.

**Supplementary Table S1**. Complete hyperparameter specification used for all four architectures across every reported run.

| **Hyperparameter** | **Value** |
| --- | --- |
| Optimizer | AdamW (Loshchilov & Hutter, 2019) |
| Learning rate | 1 × 10⁻³ |
| Weight decay | 1 × 10⁻⁴ |
| LR schedule | CosineAnnealingLR, *T_max_* = 50, *η_min_* = 1 × 10⁻⁶ |
| Gradient clipping | global ℓ₂-norm = 1.0 |
| Mini-batch size | 32 (Transformer / InceptionTime / LSTM-CNN); 8 (ShapeFormer) |
| Max epochs | 200, early stop patience = 20 on validation accuracy |
| Loss (primary) | class-weighted CE; weights = *N_train_* / (*C* · *n_c_*), participant level |
| Loss (sensitivity) | focal loss *γ* = 2.0, same class weights |
| CV protocol | Stratified, participant-level. 80/20 holdout (33 reserved); 10-fold StratifiedKFold on the 80% development set; all 10 folds reported (val ≈ 13) |
| CV seed (fixed) | 1 (matching original main.py default) |
| Model seeds | 123, 456, 789 — three seeds × ten folds = 30 paired observations per model |
| Search strategy | No formal hyperparameter search; values fixed from initial debugging on training partitions only |

**Supplementary Table S2**. Friedman omnibus and Holm-corrected paired t-tests across the ten participant-level folds (seeds collapsed to per-fold averages). *n* = 10 paired observations per pairwise comparison.

|  | **Friedman *χ²*(3)** | ***p*-value** | **Holm-corrected pairwise sig.** |
| --- | --- | --- | --- |
| Accuracy | 7.80 | 0.050 | none |
| Macro F1-score | 7.56 | 0.056 | none |
| AUC-macro | 0.84 | 0.840 | none |
| Frail-class recall | 3.78 | 0.286 | none |
| Frail-class F1-score | 3.95 | 0.267 | none |

**Supplementary Table S3**. Focal loss vs class-weighted cross-entropy, 30 runs per (model × criterion) under the identical CV protocol.

|  | **Loss** | **Accuracy** | **Macro F1-score** | **Frail-class**  **F1-score** |
| --- | --- | --- | --- | --- |
| Transformer | weighted CE | 72.6 ± 6.0 | 68.4 ± 12.6 | 56.8 ± 37.2 |
|  | focal (*γ*=2) | 70.8 ± 10.6 | 65.8 ± 15.3 | 52.7 ± 37.9 |
| InceptionTime | weighted CE | 70.5 ± 9.5 | 70.3 ± 12.8 | 73.0 ± 29.5 |
|  | focal (*γ*=2) | 69.5 ± 8.9 | 69.7 ± 10.4 | 73.3 ± 22.4 |
| LSTM-CNN | weighted CE | 65.9 ± 9.8 | 64.5 ± 11.6 | 63.3 ± 24.1 |
|  | focal (*γ*=2) | 63.6 ± 8.6 | 62.6 ± 9.4 | 65.5 ± 22.1 |
| ShapeFormer | weighted CE | 73.1 ± 10.6 | 71.5 ± 12.2 | 70.2 ± 26.3 |
|  | focal (*γ*=2) | 73.8 ± 10.6 | 70.5 ± 13.4 | 63.7 ± 30.2 |

**Supplementary Table S4**. Frail-class precision-recall summary and threshold-tuned operating points per architecture (mean ± SD across the 30 outer-fold operating points).

|  | **Average precision (frail-class)** | **Default-threshold**  **P / R** | **Precision ≥ 0.50:**  **P / R** |
| --- | --- | --- | --- |
| Transformer | 0.47 | 60 ± 41 / 62 ± 41 | 62 ± 22 / 65 ± 42 |
| InceptionTime | 0.74 | 72 ± 33 / 82 ± 33 | 55 ± 15 / 85 ± 33 |
| LSTM-CNN | 0.52 | 55 ± 28 / 85 ± 27 | 53 ± 13 / 82 ± 31 |
| ShapeFormer | 0.60 | 68 ± 31 / 82 ± 31 | 53 ± 13 / 83 ± 30 |


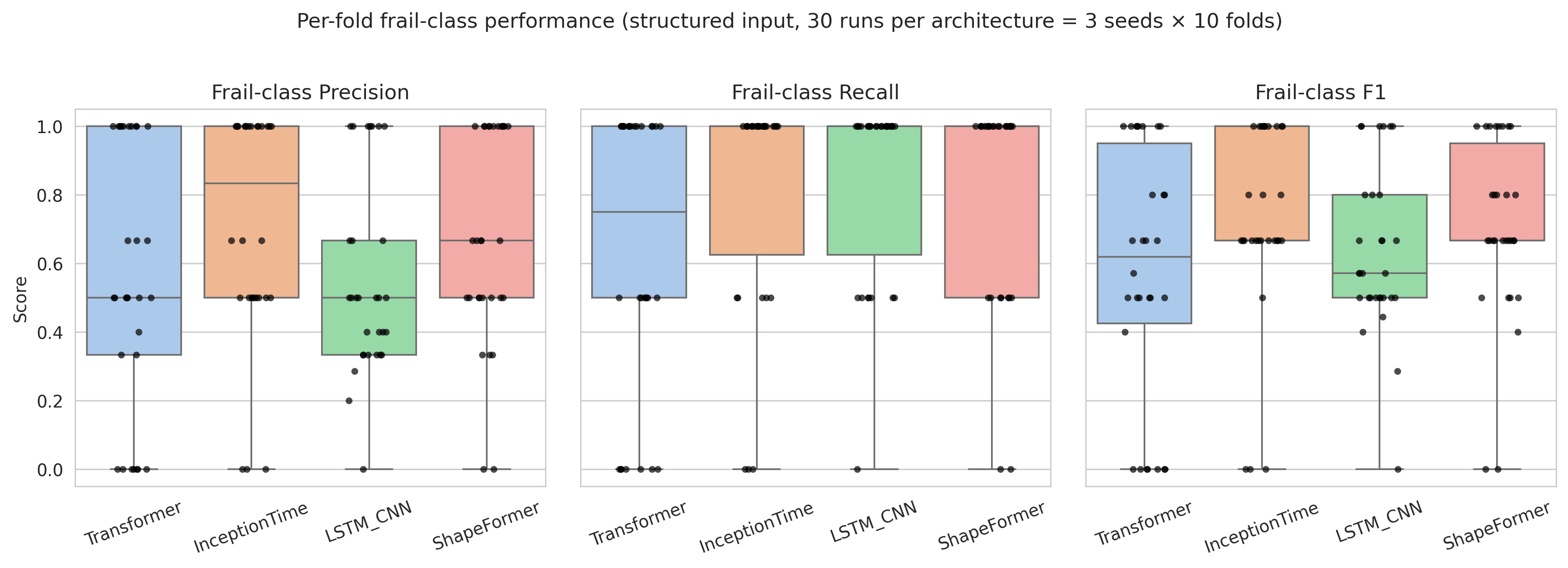


**Supplementary Figure S1**. Per-fold frail-class precision / recall / F1 across the 30 (3 seeds × 10 folds) runs per architecture, structured features.


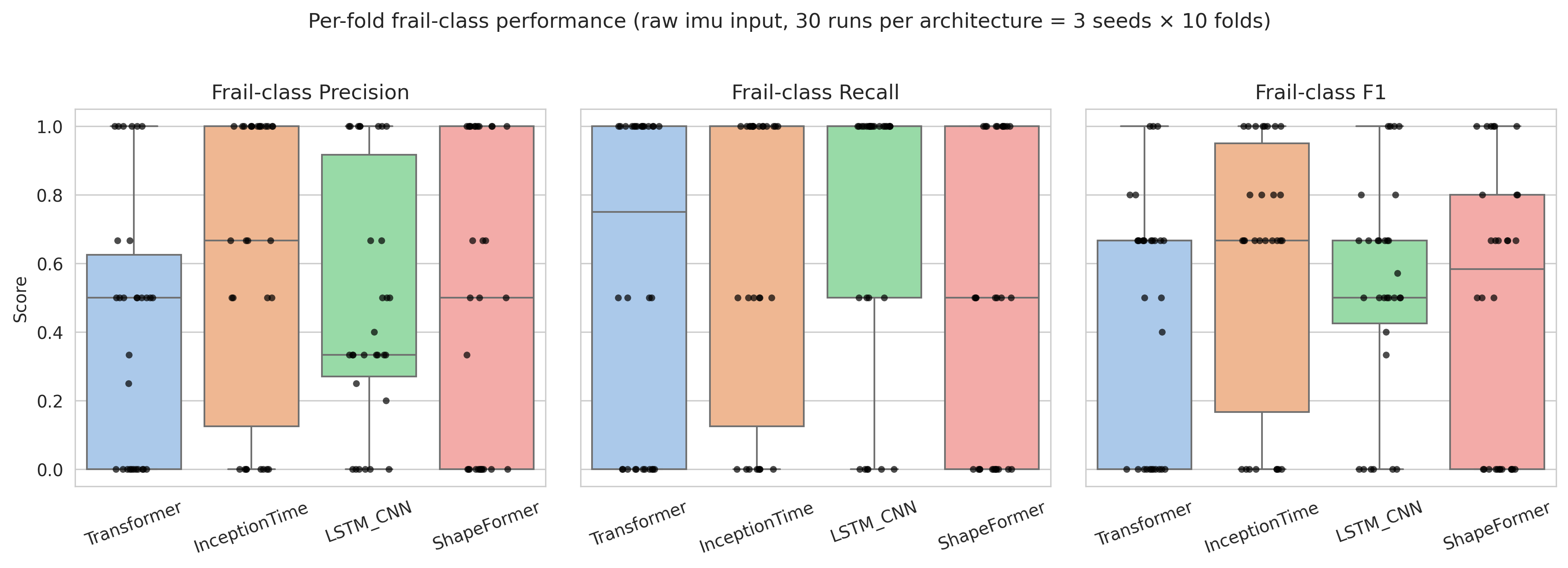


**Supplementary Figure S2**. Same as Supplementary Figure S1 but for raw IMU input (*n* = 158 participants). ShapeFormer was retrained on raw IMU using its internal resampling pipeline, allowing direct comparison with the other architectures.


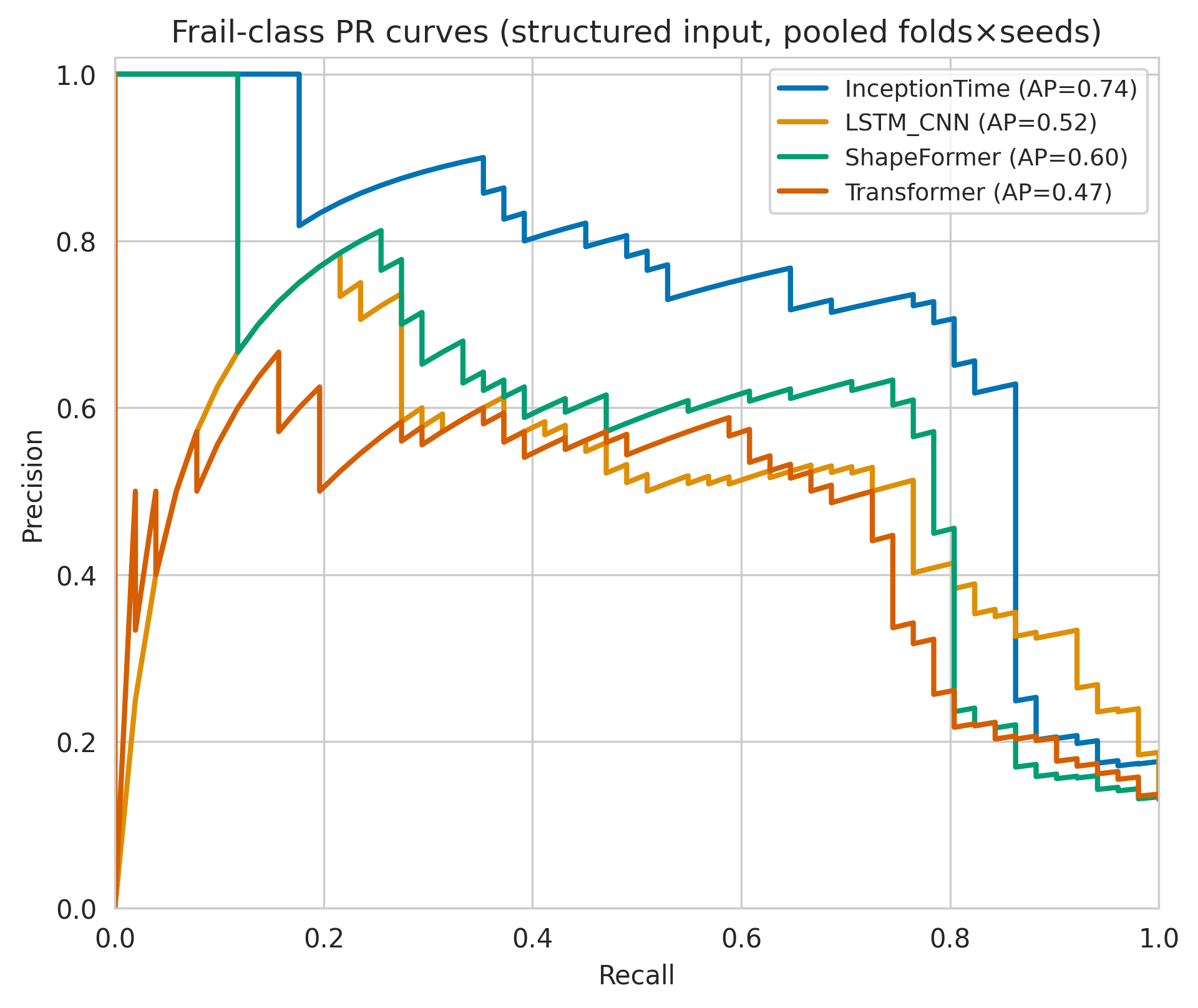


**Supplementary Figure S3**. Frail-class precision-recall curves under focal loss, pooled across the 30 (seed × fold) runs per model. Average precision (AP) is shown in the legend; random baseline = 0.13.
